# Identification of long non-coding RNAs and RNA binding proteins in breast cancer subtypes

**DOI:** 10.1038/s41598-021-04664-z

**Published:** 2022-01-13

**Authors:** Claudia Cava, Alexandros Armaos, Benjamin Lang, Gian G. Tartaglia, Isabella Castiglioni

**Affiliations:** 1 Institute of Molecular Bioimaging and Physiology, National Research Council (IBFM-CNR), Via F.Cervi 93, 20090 Segrate-Milan, Milan, Italy; 2 Centre for Genomic Regulation (CRG), The Barcelona Institute for Science and Technology, C/ Dr. Aiguader 88, 08003 Barcelona, Spain; 3 RNA System Biology Lab, Department of Neuroscience and Brain Technologies, Istituto Italiano Di Tecnologia (IIT), Via Morego 30, 16163 Genoa, Italy; 4 Department of Structural Biology and Center for Data Driven Discovery (C3D), St. Jude Children’s Research Hospital, Memphis, TN 38105 USA; 5 Sapienza University of Rome, Piazzale Aldo Moro 5, 00185 Rome, Italy; 6 Department of Physics “Giuseppe Occhialini”, University of Milan-Bicocca Piazza dell’Ateneo Nuovo, 1 - 20126, Milan, Italy

**Keywords:** Computational biology and bioinformatics, Molecular biology

## Abstract

Breast cancer is a heterogeneous disease classified into four main subtypes with different clinical outcomes, such as patient survival, prognosis, and relapse. Current genetic tests for the differential diagnosis of BC subtypes showed a poor reproducibility. Therefore, an early and correct diagnosis of molecular subtypes is one of the challenges in the clinic. In the present study, we identified differentially expressed genes, long non-coding RNAs and RNA binding proteins for each BC subtype from a public dataset applying bioinformatics algorithms. In addition, we investigated their interactions and we proposed interacting biomarkers as potential signature specific for each BC subtype. We found a network of only 2 RBPs (RBM20 and PCDH20) and 2 genes (HOXB3 and RASSF7) for luminal A, a network of 21 RBPs and 53 genes for luminal B, a HER2-specific network of 14 RBPs and 30 genes, and a network of 54 RBPs and 302 genes for basal BC. We validated the signature considering their expression levels on an independent dataset evaluating their ability to classify the different molecular subtypes with a machine learning approach. Overall, we achieved good performances of classification with an accuracy >0.80. In addition, we found some interesting novel prognostic biomarkers such as RASSF7 for luminal A, DCTPP1 for luminal B, DHRS11, KLC3, NAGS, and TMEM98 for HER2, and ABHD14A and ADSSL1 for basal. The findings could provide preliminary evidence to identify putative new prognostic biomarkers and therapeutic targets for individual breast cancer subtypes.

## Introduction

Breast cancer (BC) is one of the most common cancers around the world and was estimated the most frequent cancer among women (25% of all new cancers recorded)^1^. The heterogeneity of BC reduces the specificity of biological features (e.g., histological grade and hormone receptor status) which are usually utilized for the diagnosis and prognosis of BC and to address a therapy^2,3^. The classification of biological BC subtypes is based on the use of techniques such as immunohistochemistry and gene expression profiling^4^.

In 2011 The St. Gallen International Breast Cancer Conference reported a molecular subtype approach to guide the therapy of BC based on immunohistochemical markers: estrogen receptor (ER), progesterone receptor (PR), and human epidermal growth factor receptor 2 (HER2)^4^. In addition to the detection of these standard biomarkers, St. Gallen in 2013 included the evaluation of a marker of cell proliferation: Ki-67^5^. Luminal A is defined by ER positive and/or PR positive and Ki-67 < 14%, and luminal B by ER positive and/or PR positive and Ki-67 ≥ 14%. ER negative, PR negative and Her2 positive tumors are classified as HER2 + ^6^. Triple negative BC (TNBC) are characterized by ER negative and PR negative and Her2 negative^6^.

The development of gene expression profiling with microarray demonstrated that the classification based on gene expression profiling reflects the differences of BC subtypes at the molecular level^3^. The pioneer study of Perou et al. in 2000 reported that BC could be classified into four intrinsic molecular subtypes by gene expression profiling: luminal A, luminal B, HER2-enriched (HER2), and basal^7,8^. Gene expression classifi-cation defines TNBC of immunohistochemistry with term basal BC. However, previous studies reported that there is a concordance of 80% between TNBC and basal BC^9^. Unlike the TNBC subtype, basal BC is characterized by the expression of other proteins, such as cytokeratins 5,6 and 17^10^.

BC molecular subtypes can be detected by different genetic tests with a different gene signature (e.g., PAM50, MammaPrint, and Oncotype DX). Several studies, applied to publicly available gene expression datasets, demonstrated a poor reproducibility among different genetic tests. This can be explained by the differences of gene signature in different genetic tests^11,12^. These observations forced the research towards the discovery of new biomarkers to be used for BC subtype characterization.

Luminal A is the most common BC subtype with a higher favorable prognosis and a slower evolution^13^. Luminal B subtype is characterized by an intermediate prognosis compared with luminal A and HER2 BC and an increased expression of genes associated with growth receptor signaling^14^. HER2 BC frequently tend to metastasize in the brain, liver and lung. In addition, the overexpression of HER2 is implicated in the cell proliferation, blocking apoptosis and cell spreading^15^. Basal BC subtype has a worse prognosis compared with other subtypes and high cell proliferation. Non-luminal tumors form metastases into distant organs more frequently than luminal tumors, but surprisingly luminal A and basal subtypes develop the regional lymph node metastases less often^16,17^. The luminal A is well differentiated compared to luminal B, HER2 and basal that are poorly differentiated^17^.

Previous studies reported that the evolution from normal breast cell types to BC subtypes derives from mutations or genetic rearrangements in stem cells and progenitor cells giving rise to a heterogeneous population of cells^18^.

New more accurate methods are needed to increase prognostic value and to personalize the most appropriate treatment for patients with BC and to investigate the molecular mechanisms responsible of BC subtypes differentiation. In the recent years Long Non-Coding RNAs (lncRNAs) and RNA binding-proteins (RBPs) emerged as key regulators of post-transcriptional events, and they are dysregulated in many human solid cancers, including BC^19,20^.

LncRNAs, longer than 200 nucleotides in length, belong to a large class of noncoding RNAs and are implicated in the regulation of gene expression by different mechanisms that are not yet fully characterized^21,22^. Previous studies observed their role in several physiological and pathological events^23^.

Because of the poor prognosis detected in BC patients and the lack of standard therapeutic treatments that avoid chemoresistance is needed the study of molecular profiling to better describe the BC subtypes with higher accuracy. This would allow the understanding of the altered molecular mechanisms in a specific subtype of BC.

Recently, several studies have observed a strong association of lncRNAs with BC development, progression, and metastasis. Basically, lncRNAs could act as promoters or inhibitors of BC cell invasion and metastasis. However, few studies reported the association between lncRNAs and molecular subtypes of BC^24^.

Notably, as lncRNAs could be found in human body fluids, the characterization of lncRNAs offer the opportunity to avoid the difficulties related with tissue biopsy of the currently genetic tests (e.g., OncotypeDX).

In addition, the importance of lncRNAs in the administration of anticancer treatment is encouraged by their involvement in drug resistance in cancer. For example, in prostate cancer, numerous lncRNAs are correlated with resistance to hormonal therapy, such as NEAT1 and PRNCR1^25,26^.

RBPs are involved in a wide range of molecular processes including cell adhesion and response to stress. Many RBPs bind to sequence-specific motifs or RNA secondary structures, or a combination of both to regulate RNA metabolism and function. RBPs participate in the generation of ribonucleoprotein complexes that are principally implicated in gene expression processes such as splicing, mRNA synthesis and degradation^27,28^. In addition, RBPs are found differentially expressed in different cancers and are able to regulate the expression of oncogenes and tumor suppressor genes^29^. Therefore, the characterization of RPBs could reveal novel targets of cancer treatment by studying the mechanisms behind RBP expression and the association between RBPs and RNAs^30^. This notwithstanding, many lncRNAs and RBPs have not yet been studied in detail^31^.

The main goal of the present study was to assess the interactions between differentially expressed genes, lncRNAs and RBPs in different BC subtypes classified by PAM50 classifier. Firstly, we identified differentially expressed genes, lncRNAs and RBPs for each BC subtype using a published dataset. Then, we studied their interactions specific for each BC subtype. Finally, we validated the interactions with a machine learning approach on an independent dataset.

## Methods

### Data

The study is applied on a BC dataset originated from The Cancer Genome Atlas (TCGA): TCGA-BRCA. In particular, we used the expression levels of mRNA, lncRNA and RBP extracted from Illumina HiSeq RNASeqV2 platform derived by 233 BC luminal A samples, 103 BC luminal B samples, 74 BC basal samples, 43 BC HER2 samples and 113 normal samples (NS). Clinical data were downloaded from TCGA and BC subtypes were previously determined by the molecular classification of 50-genes (PAM 50 predictor)^32^. We used TCGABiolinks package 2.18^33^ to download RNAseq-data of BC subtypes and to estimate differentially expressed genes between BC subtypes and normal tissues.

**Figure 1:**
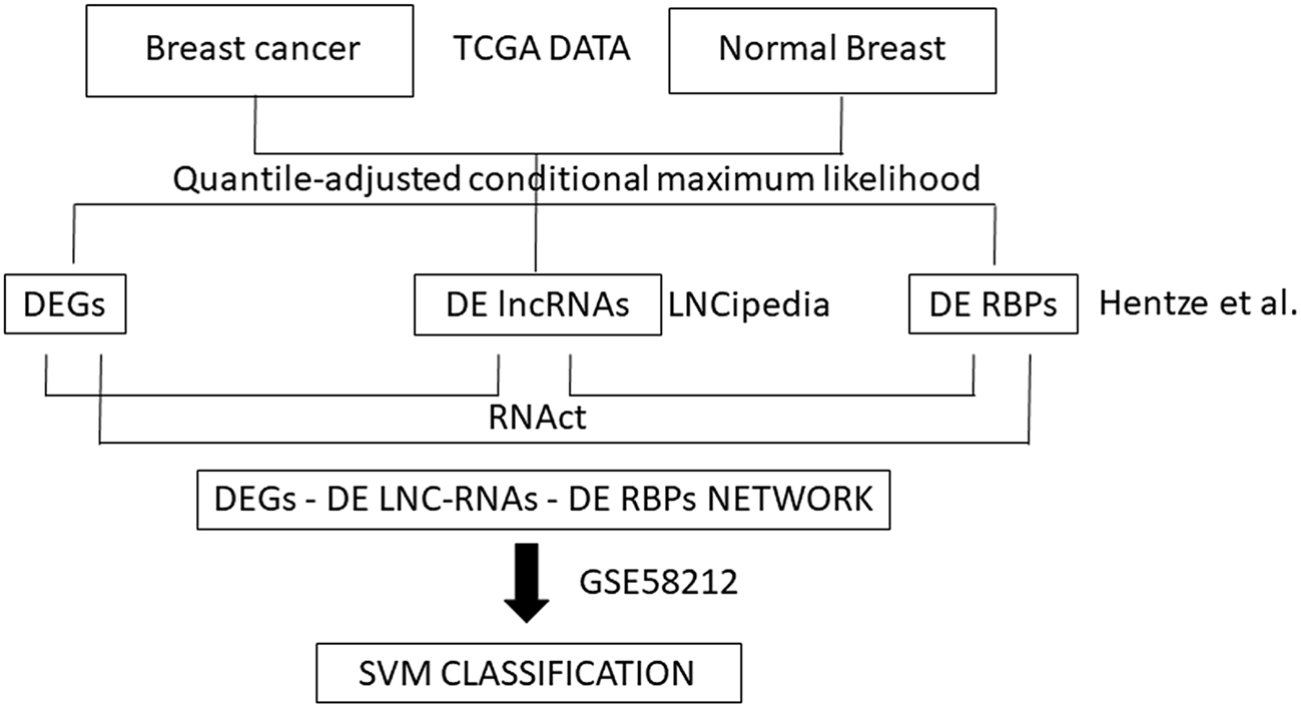
Workflow of the computational approach. The computational method was applied considering the comparison of each breast cancer (BC) subtype vs normal breast tissue. Differential expressional analysis with quantile-adjusted conditional maximum likelihood was performed on The Cancer Genome Atlas (TCGA) data between each breast cancer subtype and normal breast tissue. The analysis identified differentially expressed genes (DEGs), differentially long non-coding RNAs (DE lncRNAs) and differentially RNA-binding proteins (DE RBPs). lncRNAs and RBPs were defined by LNCipedia and by study of Hentze et al. Furthermore, we evaluated the interactions among DEGs, DE lncRNAs and DE RBPs using RNAct tool. The expression levels of interacting DEGs, DE lncRNAs and DE RBPs specific for each BC subtype were considered as biomarkers to classify BC subtypes with Support Vector Machine (SVM) classification, using an independent dataset of Gene Expression Omnibus (GEO), GSE58212.

Figure 1 shows the workflow of the computational approach.

### Differential expression analysis

Quantile-adjusted conditional maximum likelihood was used as statistical test to detect differentially expressed RNAs between normal breast tissue and BC^32^. This analysis was performed considering each BC subtype at time compared to normal breast tissue. Those genes with a |log2(fold change)|≥ 1 and adjusted *p*-value < 0.05 were considered to have statistical significance. The *p*-values were adjusted using the Benjamini–Hochberg procedure for multiple testing correction^34^.

We further identified significantly dysregulated RBPs based on RBP catalog of Hentze et al.^27^. To identify lncRNAs present in the RNA-seq data we consulted Ensembl Biomart 100^35^ and LNCipedia database version 5.2^36^.

Venn Diagram among RNAs in the four BC subtypes was represented using the R-package VennDiagram 1.6.20^37^.

The functional role of differentially expressed lncRNAs was investigated using Cancer lncRNA Census 2.0^38^.

### Protein-RNA interaction predictions

Protein-RNA networks were retrieved from the RNAct database^39^ containing predicted and experimentally validated^40,41^ protein-RNA interactions (which is based on UniProt release 2017_10 and GENCODE release 27). Within RNAct, the binding abilities were predicted using the catRAPID algorithm^3–44^. A z-score is calculated based on the values for experimentally determined protein-RNA interactions from eCLIP data from the ENCODE project and provides a score for the interaction of a protein-RNA pair of interest (for more details^39^).

### Survival analysis

Survival analysis was performed in October 2021 from The Human Protein Atlas website^45^. Kaplan–Meier analysis investigated the prognosis of BC patients and the differences between the survival curves were explored with the log-rank tests^46^. We considered a gene/RBP to be prognostic if *p*-value < 0.05.

### Machine learning approach

We identified subtype-specific networks and we validated with a machine learning approach their ability to classify the four BC molecular subtypes. The performances of RNA interactions for each BC subtype were evaluated with a linear support vector machine (SVM) and random forest classifiers, using the R-package caret 6.0.86^47^. We used default parameters for linear SVM and random forest classifiers.

RNA expression levels were normalized to make their scale comparable with the caret function “preprocess”. We used an independent GEO dataset (GSE58212) that includes: 121 luminal A, 69 luminal B, 32 HER2, and 36 basal samples.

## Results

### Differentially expressed long non-coding RNAs

By comparing the four breast cancer subtypes to their respective normal tissues, we found 3199 differentially expressed genes (DEGs) from the comparison “Luminal A vs. NS”, 4074 from “luminal B vs. NS”, 4134 from “HER2 vs. NS”, and 4181 from “basal vs. NS”. Supplementary file 1 shows the list of DEGs for each subtype. We used normal breast tissue as reference, as the aim of this study is the identification of biomarkers that could explain the molecular mechanisms that are implicated in the differentiation of BC subtypes from normal breast tissue.

In total, we found 19 unique lncRNAs. We obtained 7 lncRNAs (HOTAIR, HCG11, HPYR1, TSIX, EMX2OS, MYCNOS, and DLEU2) in luminal A (4 up-regulated lncRNAs and 3 down-regulated lncRNAs), 11 lncRNAs (SNHG5, PVT1, HCG11, HOTAIR, HPYR1, DLEU2, MYCNOS, LOH12CR2, EMX2OS, UCA1,and PART1) in luminal B (6 up-regulated lncRNAs and 5 down-regulated lncRNAs), 11 lncRNAs (SNHG8, XIST, SNHG5, GAS5, MYCNOS, HOTAIR, HCG11, UCA1, EMX2OS, LOH12CR2, and EGOT) in HER2 (3 up-regulated lncRNAs and 8 down-regulated lncRNAs), and 11 lncRNAs (XIST, UCA1, HCP5, MYCNOS, HOTAIR, SNHG3, MIR155HG, PART1, DLEU2, EMX2OS, and EGOT) in basal (8 up-regulated lncRNAs and 3 down-regulated lncRNAs). Table 1 shows differentially expressed long non-coding for each BC subtype.

**Table 1:** Differentially expressed long non-coding (LNC) RNAs in luminal A, luminal B, HER2 and basal breast cancer.

LumA vs normal			LumB vs normal			HER2 vs normal			Basal vs normal		
LNC	lgFC	FDR	LNC	lgFC	FDR	LNC	lgFC	FDR	LNC	lgFC	FDR
HOTAIR	2.4	2.6E-65	SNHG5	-1.2	2.4E-14	SNHG8	-1.2	1.5E-09	XIST	-1.2	6.7E-12
HCG11	-1.1	6.4E-19	PVT1	1.6	1.3E-25	XIST	-1.0	9.3E-07	UCA1	4.7	2E-156
HPYR1	2.2	1.0E-53	HCG11	-1.5	2.1E-21	SNHG5	-1.1	5.0E-08	HCP5	1.1	1.2E-10
TSIX	-1.0	9.2E-17	HOTAIR	1.9	3.7E-36	GAS5	-1.0	4.5E-07	MYCNOS	3.0	1.5E-72
EMX2OS	-1.7	3.9E-45	HPYR1	2.5	5.3E-56	MYCNOS	4	3.4E-112	HOTAIR	2.1	1.6E-37
MYCNOS	1.5	7.5E-29	DLEU2	1.8	3.6E-33	HOTAIR	2.7	4.3E-55	SNHG3	1.2	2.8E-12
DLEU2	1.3	2.0E-22	MYCNOS	1.7	2.2E-29	HCG11	-1.4	2.1E-11	MIR155HG	1.2	1.0E-13
			LOH12CR2	-1.0	3.0E-11	UCA1	2.4	2.7E-42	PART1	1.1	1.7E-11
			EMX2OS	-2.4	1.6E-50	EMX2OS	-2.2	6.6E-25	DLEU2	1.7	1.2E-23
			UCA1	1.1	1.7E-13	LOH12CR2	-1.0	4.8E-07	EMX2OS	-2.0	2.3E-29
			PART1	-1.9	6.9E-35	EGOT	-1.6	2.1E-14	EGOT	-2.2	1.4E-33

*LgGC* log-fold change, *FDR* false discovery rate.

**Figure 2:**
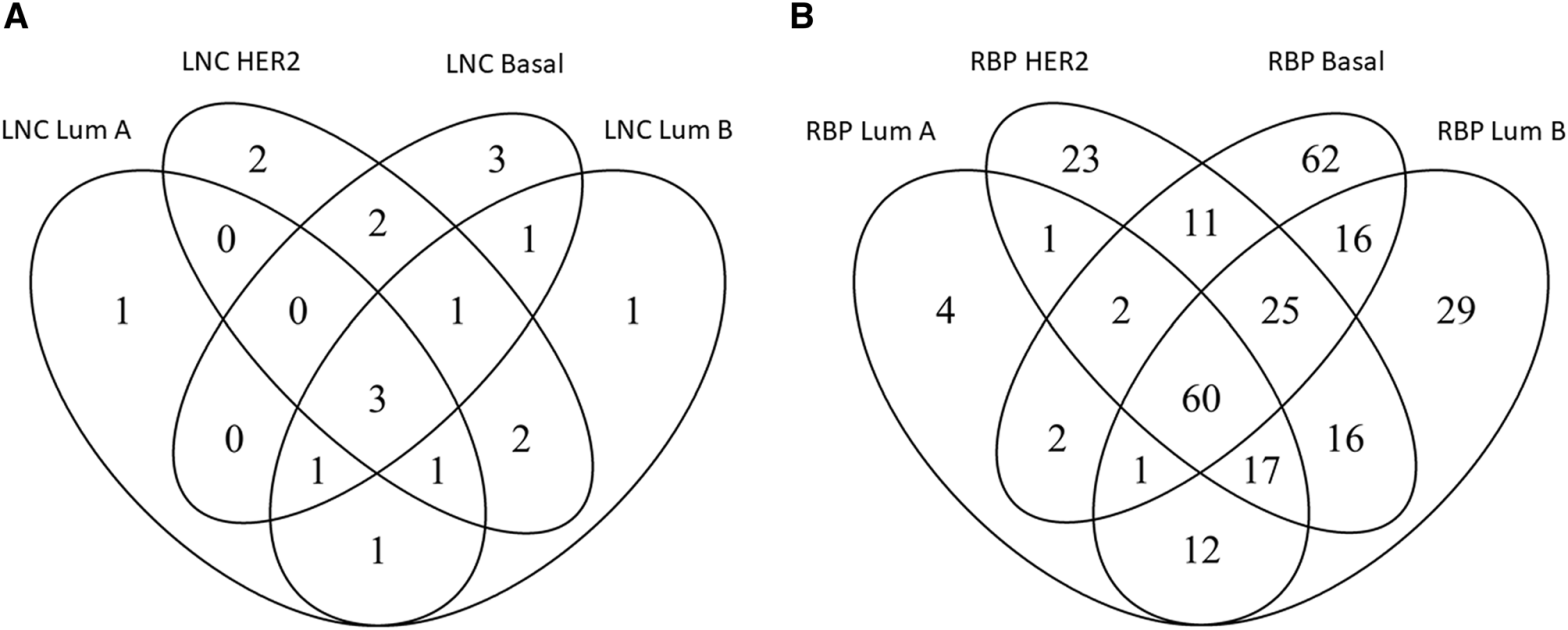
Venn diagram. It identifies: (**A**) common differentially expressed long non-coding RNAs (lncRNAs) and (**B**) RNA-binding proteins (RBPs) among breast cancer molecular subtypes.

1 lncRNA was found only in luminal A (TSIX), 1 lncRNA was found only in luminal B (PVT1), 2 lncRNAs were found only in HER2 (SNHG8 and GAS5), and 3 lncRNAs were found only in basal (HCP5, SNHG3 and MIR155HG). 3 lncRNAs were found in common among 4 BC subtypes (HOTAIR, EMX2OS and MYCNOS). Figure 2A shows a Venn diagram representing common lncRNAs among BC subtypes and subtype-specific lncRNAs.

Table 2 shows the functional information of lncRNAs extracted by Cancer lncRNA Census 2.0. We found that 14 of 15 lncRNAs were previously associated with different cancer types. GAS5, PVT1, and XIST showed a dual role as tumor suppressors and oncogenes. DLEU2 and EGOT play a role in cancer as tumor suppressors. HCG11, HCP5, HOTAIR, MYCNOS-01, PART1, SNHG3, SNHG5, SNHG8, and UCA1 were oncogenes in different caner types. To date, MIR155HG is not associated with a clear function with cancer.

**Table 2:** Functional information of lncRNAs as reported the Cancer lncRNA Census 2.0.

	GENCODE ID	Cancer function	Cancer type
DLEU2	ENSG00000231607	T	Blood; head_and_neck; liver
EGOT	ENSG00000235947	T	**Breast**; stomach; neuroepithelial; kidney
GAS5	ENSG00000234741	Both	**Breast**; kidney; prostate; pancreas; urothelial; lung; stomach; liver; colon; cervical; ovarian; endometrial; urothelial; esophageal; neuroepithelial; bone; skin; sarcoma
HCG11	ENSG00000228223	O	Liver
HCP5	ENSG00000206337	O	Thyroid;bone;cervical
HOTAIR	ENSG00000228630	O	Cervical; ovarian; blood; colon; kidney; nasopharyngeal; lung; head_and_neck; **breast**; neuroepithelial; liver; stomach; thyroid; oral; urothelial; bone; gallbladder; esophageal; endometrial; skin; pancreas; prostate; retinoblastoma; larynx
MIR155HG	ENSG00000234883	N/A	Blood
MYCNOS-01	ENSG00000233718	O	Neuroepithelial
PART1	ENSG00000152931	O	Prostate; esophageal
PVT1	ENSG00000249859	Both	Ovarian; **breast**; colon; lung; skin; kidney; prostate; liver; neuroepithelial; urothelial; stomach; pancreas; bone; esophageal; cervical; blood; thyroid; nasopharyngeal; endometrial; head_and_neck
SNHG3	ENSG00000242125	O	Colon
SNHG5	ENSG00000203875	O	Urothelial; colon; stomach
SNHG8	ENSG00000269893	O	Stomach; lung; liver
UCA1	ENSG00000214049	O	Urothelial; liver; **breast**; lung; bone; bile_duct; stomach; oral; neuroepithelial; gallbladder; kidney; cervical; prostate; head_and_neck; pancreas; blood; esophageal; endometrial; skin; ovarian; tongue; colon
XIST	ENSG00000229807	Both	Neuroepithelial; blood; lung; **breast**; pancreas; bone; urothelial; prostate; colon; esophageal; stomach; liver; nasopharyngeal; ovarian; thyroid

*T* tumor suppressors, *O* oncogenes.

### Differentially expressed RNA binding-proteins

We considered 1393 RBPs curated by Hentze et al.^27^ and identified significantly dysregulated RBPs based on the differential expression analysis as described above.

Among the 3199 differentially expressed genes in luminal A, there were 99 out of 1393 RBPs, among the 4074 DEGs in luminal B there were 176 RBPs. 155 RBPs out of 4134 DEGs were found in HER2 and among the 4181 DEGs there were 179 RBPs. Table 3 summarizes the results of differential expression analyses considering long non-coding RNAs and RNA-binding proteins compared to normal tissues.

**Table 3:** The table shows differentially expressed genes (DEGs), differentially expressed long Non-Coding (DE-lncRNAs) RNAs and differentially RNA binding-proteins (DE-RBPs).

	DEGs	DEGs Ω DE- lncRNAs	DEGs Ω DE-RBPs
233 lumA vs 113 normal	3199	7	99
103 lumB vs 113 normal	4074	11	176
43 HER2 vs 113 normal	4134	11	155
74 Basal vs 113 normal	4181	11	179
Tot (unique)	5980	19	281

4 RBPs (NUDT16L1, RBM20, PCDH20, and PCBP3) were found only in luminal A, 29 RBPs were found only in luminal B, 23 RBPs were found only in HER2, and 62 RBPs were found only in basal (Fig. 2B). Supplementary file 2 shows the list of RBPs for each subtype.

### Interactions of RNA-binding proteins

Overall, from the differential expression analysis we found 5980 unique genes for all subtypes, 19 unique lncRNAs, and 281 unique RBPs.

We obtained interaction predictions for the 5980 RNAs and 281 RBPs from the RNAct protein–RNA interaction database. Interaction predictions are prioritized by a normalized score (z-score). The distribution of z-scores in our data is shown in the Supplementary Fig. 3. We selected the protein-RNA interactions that obtained a z-score ≥ 2 since this should provide a good balance between sensitivity and specificity. We obtained a network of 2585 nodes (241 RBPs, 7 lncRNAs, and 2337 RNAs), altered in at least one BC subtype, with 45,727 interactions. Supplementary file 4 shows these interactions.

We selected direct interactions involving only differentially expressed RBPs and genes present in a single subtype, namely, we considered the subtype-specific interactions. For luminal A we found a network consisted of 2 RBPs (RBM20 and PCDH20) and 2 genes (HOXB3 and RASSF7). The specific network for luminal B includes 21 RBPs and 53 DEGs. The specific network for HER2 includes 14 RBPs and 30 DEGs. The specific network for basal includes 54 RBPs and 302 DEGs. However, we did not find lncRNAs specific for a BC subtype interacting with the RBPs and DEGs specific for the same BC subtype.

**Figure 3:**
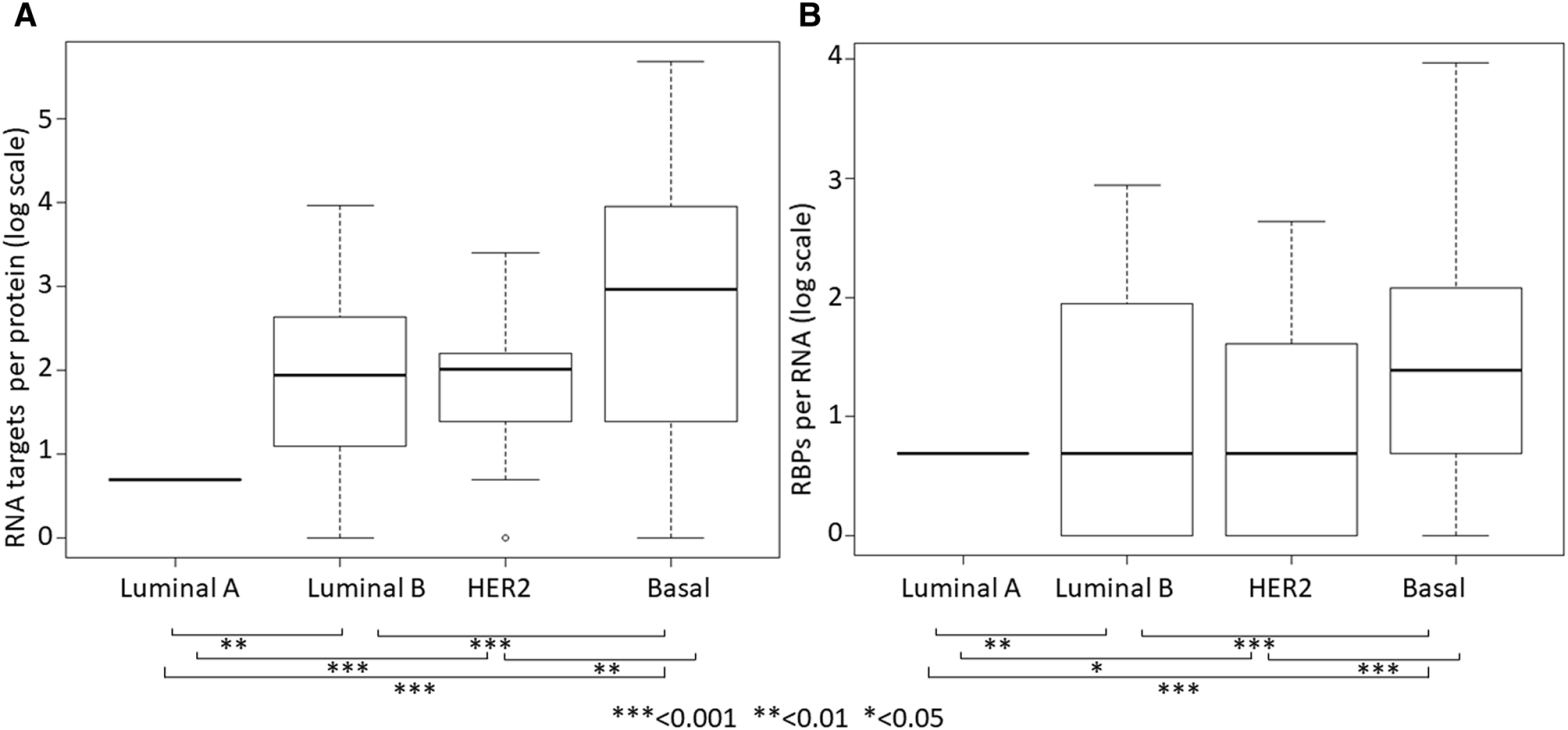
The boxplots show the number of RNAs or RNA-binding proteins (RBPs) that there are per protein or RNA, considering direct interactions involving only differentially expressed RBPs and genes present in a single breast cancer subtype (*t*-test, * *p*-value < 0.05, ** *p*-value < 0.01, *** *p*-value < 0.001).

From the subtype-specific interactions we evaluated the number of RNA targets for each RBP and the number of RBP for each RNA (Fig. 3).

We found that dysregulated RNAs in luminal A have a lower number of differentially expressed RBPs than dysregulated RNAs in luminal B, HER2 and basal BC (*p*-value < 0.001). We can explain these results indicating that the abnormal expression of RBPs increase with the worsening of the prognosis. In addition, the number of altered RNAs regulated by each altered RBP is directly proportional to the aggressiveness of the BC subtype, suggesting RBP as potential biomarkers responsible of BC subtypes differentiation.

We analyzed the prognostic role of subtype-specific RBPs and genes (Table 4). We found that 43% of specific RBPs for luminal B, 57% of specific RBPs for HER2, and 46% of specific RBPs for basal can differentiate BC patients with good and poor prognosis. 50% of specific genes for luminal A, 51% of specific genes for luminal B, 40% of specific genes for HER2, and 40% of specific genes for basal may influence BC patient survival.

**Table 4:** List of potential prognostic subtype specific RBPs/genes as obtained from The Human Protein Atlas.

	Prognostic RBP	Prognostic gene
Luminal A		RASSF7 (*p*-value = 0.01)
Luminal B	CSE1L (*p*-value = 0.04) HOOK1 (*p*-value = 0.009) KIF1C (*p*-value = 0.03) LSM1 (*p*-value = 0.01) MRPS23 (*p*-value = 0.003) MTDH (*p*-value = 0.001) UTP18 (*p*-value = 0.01) YBX2 (*p*-value = 0.01) ZFP36L1 (*p*-value = 0.01)	ABHD12 (*p*-value = 0.00003) ANAPC11 (*p*-value = 0.009) ASIP (*p*-value = 0.04) CCDC107 (*p*-value = 0.003) CLYBL (*p*-value = 0.01) COMMD3 (*p*-value = 0.03) COMMD5 (*p*-value = 0.02) CYC1 (*p*-value = 0.03) DCTPP1 (*p*-value = 0.00001) DUSP22 (*p*-value = 0.007) EPB41L4B (*p*-value = 0.0001) ETS1 (*p*-value = 0.01) FDXR (*p*-value = 0.02) GRINA (*p*-value = 0.002) HYI (*p*-value = 0.0009) IL1R1 (*p*-value = 0.006) ITM2C (*p*-value = 0.002) JDP2 (*p*-value = 0.03) LBH (*p*-value = 0.02) LYPLA1 (*p*-value = 0.02) OLFM2 (*p*-value = 0.01) PHF20L1 (*p*-value = 0.009) PLEKHF1 (*p*-value = 0.05) SDC3 (*p*-value = 0.001) SYNGR2 (*p*-value = 0.02) TSEN54 (*p*-value = 0.03) UBE2L6 (*p*-value = 0.0006)
HER2	ALDH18A1 (*p*-value = 0.006) CXorf57 (*p*-value = 0.03) HSPD1 (*p*-value = 0.02) PRDX1 (*p*-value = 0.01) RPL22 (*p*-value = 0.003) RPL26 (*p*-value = 0.02) SBDS (*p*-value = 0.0001) SPATS2 (*p*-value = 0.02)	ARRDC1 (*p*-value = 0.03) CHPT1 (*p*-value = 0.001) CITED4 (*p*-value = 0.005) DHRS11 (*p*-value = 0.002) FBXO2 (*p*-value = 0.02) KLC3 (*p*-value = 0.04) NAGS (*p*-value = 0.001) PKIG (*p*-value = 0.03) PPP4R4 (*p*-value = 0.002) RAB40B (*p*-value = 0.02) SMARCD3 (*p*-value = 0.01) TMEM98 (*p*-value = 0.01)
Basal	BTF3 (*p*-value = 0.003) BYSL (*p*-value = 0.006) CCT6A (*p*-value = 0.0004) CORO1A (*p*-value = 0.004) CPEB3 (*p*-value = 0.04) DKC1 (*p*-value = 0.009) EIF4B (*p*-value = 0.01) ENO1 (*p*-value = 0.006) EPRS (*p*-value = 0.0007) FASN (*p*-value = 0.03) GTPBP4 (*p*-value = 0.002) HDAC2 (*p*-value = 0.0007) ILF2 (*p*-value = 0.002) IQCG (*p*-value = 0.01) LARP4B (*p*-value = 0.003) MAGOH (*p*-value = 0.0001) MAGOHB (*p*-value = 0.01) MRPL15 (*p*-value = 0.005) NDRG1 (*p*-value = 0.007) NPM3 (*p*-value = 0.03) NSA2 (*p*-value = 0.004) RPS14 (*p*-value = 0.005) SNRPD1 (*p*-value = 0.01) UCHL5 (*p*-value = 0.03) YARS (*p*-value = 0.04)	ABCD4 (*p*-value = 0.03) ABHD14A (*p*-value = 0.004) ACOT1 (*p*-value = 0.004) ACOT2 (*p*-value = 0.004) ACSF2 (*p*-value = 0.01) ADRA2C (*p*-value = 0.01) ADSSL1 (*p*-value = 0.02) APBB2 (*p*-value = 0.04) ASNS (*p*-value = 0.03) C2CD4D (*p*-value = 0.008) CAMK2B (*p*-value = 0.001) CAPG (*p*-value = 0.02) CCDC103 (*p*-value = 0.009) CCDC28B (*p*-value = 0.0002) CCNG1 (*p*-value = 0.02) CD6 (*p*-value = 0.0002) CD7 (*p*-value = 0.003) CD72 (*p*-value = 0.01) CD8B (*p*-value = 0.001) CDC123 (*p*-value = 0.005) CGREF1 (*p*-value = 0.01) CKS1B (*p*-value = 0.01) COQ10A (*p*-value = 0.04) COTL1 (*p*-value = 0.02) CRADD (*p*-value = 0.01) CROT (*p*-value = 0.02) CTDSP1 (*p*-value = 0.02) CTSF (*p*-value = 0.01) DOK3 (*p*-value = 0.02) EMID1 (*p*-value = 0.02) EPHX1 (*p*-value = 0.02) FAM117A (*p*-value = 0.00005) FAM172A (*p*-value = 0.01) FAM189B (*p*-value = 0.01) FAM24B (*p*-value = 0.04) FAM26F (*p*-value = 0.01) FOXQ1 (*p*-value = 0.002) FRAT1 (*p*-value = 0.01) FSTL3 (*p*-value = 0.009) FXYD7 (*p*-value = 0.001) GHDC (*p*-value = 0.02) GJB3 (*p*-value = 0.001) GJC1 (*p*-value = 0.01) GNB4 (*p*-value = 0.02) GNMT (*p*-value = 0.01) GPX4 (*p*-value = 0.008) GRP (*p*-value = 0.03) GSC (*p*-value = 0.02) GSTM3 (*p*-value = 0.02) HHEX (*p*-value = 0.0008) HLA-B (*p*-value = 0.02) HLA-DQB1 (*p*-value = 0.008) HLA-DRB1 (*p*-value = 0.003) HLA-F (*p*-value = 0.003) HLA-G (*p*-value = 0.01) HSD17B8 (*p*-value = 0.001) HTATIP2 (*p*-value = 0.008) HYOU1 (*p*-value = 0.03) ICAM1 (*p*-value = 0.0007) IFITM2 (*p*-value = 0.02) IL12RB1 (*p*-value = 0.007) IL2RG (*p*-value = 0.003) IL32 (*p*-value = 0.02) IRAK1 (*p*-value = 0.004) ISG20 (*p*-value = 0.002) KRTCAP3 (*p*-value = 0.00002) LCK (*p*-value = 0.006) LRRC14B (*p*-value = 0.0007) LYPD5 (*p*-value = 0.004) MAP4K1 (*p*-value = 0.002) MDFI (*p*-value = 0.009) MORN1 (*p*-value = 0.001) MTHFD1L (*p*-value = 0.03) NDUFB9 (*p*-value = 0.01) NFKBIE (*p*-value = 0.00001) NFKBIZ (*p*-value = 0.02) OPN3 (*p*-value = 0.02) PCDHB2 (*p*-value = 0.002) PDK3 (*p*-value = 0.005) PEX11G (*p*-value = 0.02) PGPEP1 (*p*-value = 0.009) PHF7 (*p*-value = 0.00007) PLA2G7 (*p*-value = 0.003) PSMB8 (*p*-value = 0.0005) PSMG1 (*p*-value = 0.01) PTS (*p*-value = 0.02) PUSL1 (*p*-value = 0.02) PVR (*p*-value = 0.002) PXMP4 (*p*-value = 0.03) QPCT (*p*-value = 0.006) RBM43 (*p*-value = 0.03) RBP1 (*p*-value = 0.009) REEP5 (*p*-value = 0.003) RELB (*p*-value = 0.0001) RHOB (*p*-value = 0.009) RNF212 (*p*-value = 0.02) RNF8 (*p*-value = 0.01) RPS6KA5 (*p*-value = 0.03) S100A3 (*p*-value = 0.03) SAP30L (*p*-value = 0.003) SETD7 (*p*-value = 0.0008) SH2B2 (*p*-value = 0.02) SHC2 (*p*-value = 0.02) SKP2 (*p*-value = 0.02) SLC26A1 (*p*-value = 0.02) SMOX (*p*-value = 0.01) SPIB (*p*-value = 0.0004) SPOCK2 (*p*-value = 0.0004) SSBP2 (*p*-value = 0.04) ST8SIA6 (*p*-value = 0.004) SULT1A1 (*p*-value = 0.02) SUV39H2 (*p*-value = 0.005) TAPBP (*p*-value = 0.0002) TARBP1 (*p*-value = 0.003) THRA (*p*-value = 0.02) TMEM135 (*p*-value = 0.01) TMEM25 (*p*-value = 0.01) TMEM52 (*p*-value = 0.04) TOMM5 (*p*-value = 0.01) TOP1MT (*p*-value = 0.005) TPSAB1 (*p*-value = 0.02) TRIM47 (*p*-value = 0.00006) TSC22D3 (*p*-value = 0.03) TSKU (*p*-value = 0.01) TSPAN33 (*p*-value = 0.04) TTC36 (*p*-value = 0.001) UPK3A (*p*-value = 0.01) ZDHHC23 (*p*-value = 0.002) ZFYVE21 (*p*-value = 0.01) ZNF582 (*p*-value = 0.001)

*P*-value is indicated as obtained from the log-rank test.

We found a lower *p*-value (*p*-value = 0.00001) for DCTPP1 and NFKBIE.

### Validation of protein-RNA interactions for each BC subtype

We validated subtype-specific interactions on an independent dataset from the NCBI Gene Expression Omnibus (GEO), GSE58212. We used the expression levels of interacting biomarkers for the classification, namely the expression levels of 2 RBPs (RBM20 and PCDH20) and 2 genes (HOXB3 and RASSF7) that we obtained for luminal A, the 21 RBPs and 53 DEGs obtained for luminal B, the 14 RBPs and 30 DEGs for HER2, and 54 RBPs and 302 DEGs obtained for basal.

Using BC subtype-specific interactions we trained (75% of the original dataset) and tested (25% of the original dataset) a classifier with a linear support vector machine (SVM) and random forest models obtaining good performances (Table 5). We achieved the best accuracy from the comparison HER2 vs other subtypes (accuracy = 0.96) and basal vs other subtypes (accuracy = 0.96) using SVM classifier. Good performances were achieved with both classifiers for luminal A vs other subtypes and basal vs other subtypes. Low sensitivity was obtained in luminal B vs other subtypes and HER2 vs other subtypes with random forest classification.

**Table 5:** Performance of classification Support Vector Machine (SVM) and Random Forest (RF) using subtype-specific interactions (sensitivity, specificity and accuracy).

	LumA vs lumB,HER2,basal		LumB vs lumA,HER2,basal		HER2 vs lumA,lumB,basal		Basal vs lumA,lumB,HER2	
	SVM	RF	SVM	RF	SVM	RF	SVM	RF
Sensitivity	0.86	0.93	0.64	0.35	0.87	0.12	0.77	0.78
Specificity	0.91	0.94	0.91	0.96	0.98	1	1	1
Accuracy	0.89	0.94	0.84	0.80	0.96	0.89	0.96	0.97

## Discussion

In this study, we examined the DEGs, differentially expressed lncRNAs and differentially expressed RBPs and their interactions in different BC subtypes. Firstly, we analyzed differentially expressed genes from the comparison luminal A vs normal samples, luminal B vs normal samples, HER2 vs normal samples, and basal vs normal samples. We obtained 3199 DEGs in luminal A, 4074 DEGs in luminal B, 4134 DEGs in HER2, and 4181 DEGs in basal.

Then, we focused on subtype-specific lncRNAs and we found 1 luminal A-specific lncRNA (TSIX), 1 luminal B-specific lncRNA (PVT1), 2 HER2-specific lncRNAs (SNHG8 and GAS5) and 3 basal-specific lncRNAs (HCP5, SNHG3 and MIR155HG), as reported in Fig. 2A.

TSIX mediates the X chromosome inactivation acting as a XIST repressor. Indeed, a strong inverse correlation between XIST and TSIX was demonstrated: a down-regulation of TSIX leads to an up-regulation of XIST that causes the inactivation of the X chromosome^48^. A previous study demonstrated that TSIX together with other lncRNAs such as OIP5-AS1, TUG1, NEAT1, MALAT1, and XIST were able to synergistically regulate cancer genes and pathways across different cancer types^49^. In addition, TSIX was differentially expressed in lung cancer^50^.

A previous study reported that PVT1 was up-regulated in BC tissues when compared with the normal tissues and its silencing repressed tumor growth^51^. PVT1 is associated with other types of cancers such as lung and ovarian cancer and is correlated with the survival of patients^52^. Furthermore, PVT1 regulates several cancers processes and pathways such ad cell–cell adhesion and TGF‐β signaling pathway. A previous study reported that PVT1 was co-expressed with another gene in the TGF‐β signaling pathway. Indeed, PVT1 can regulate the protein stability of the MYC oncogenic protein^53^.

In our study, 2 HER2-specific lncRNAs (SNHG8 and GAS5) were found. SNHG8 was overexpressed in different types of cancer, suggesting its role in the progression of these tumors. The silencing of SNHG8 inhibits the proliferation and invasion of BC cells, MCF-7 and ZR-75-30^54^. A recent study showed a possible molecular mechanism of action of SNHG8: it is a sponge for miR-656-3p and modulates SATB1 expression. SATB1 is associated with cancer cell proliferation, migration, and invasion^55^.

GAS5 is a strong candidate as prognostic biomarker since it was identified significantly downregulated in BC and correlated with poor prognosis. In addition, GAS5 is also a potential drug target since it is implicated in resistance to multiple drugs in BC such as tamoxifen and lapatinib^56^.

3 basal-specific lncRNAs (HCP5, SNHG3 and MIR155HG) were found. HCP5 was positively associated with the expression of immune checkpoints since it is mainly found expressed in immune system cells. In addition, HCP5 promotes tumor growth in vivo and in vitro as well as apoptosis and proliferation^57^. A recent study suggested that HCP5 could be a promising drug target in triple negative BC^58^.

The oncogene SNHG3 was up-regulated in BC cells and is associated with the growth of cell proliferation regulating tRNA processing and signal transduction. A recent study suggested that the down-regulation of SNHG3 might act as a possible therapeutic strategy for BC. In addition, it was demonstrated an inverse correlation between SNGH3 and *miR-154-5p*: increase of SNHG3 inhibits *miR-154-5p* and upregulates BC cell proliferation^59^.

Few is known about the role of MIR155HG in BC. MIR155HG is a precursor of *miR-155-5p* and was identified as a direct target of FOX3^40^. A recent study proposed the study of the expression MIR155HG together with FANCI and C-MYC as potential diagnostic test and drug targets in gynaecological malignancies^60^.

Then, in our study we focused on subtype-specific RBPs. 4 RBPs were found only in luminal A, 29 RBPs were found only in luminal B, 23 RBPs were found only in HER2, and 62 RBPs were found only in basal (Fig. 2B).

We found NUDT16L1, RBM20, PCDH20, and PCBP3 as luminal A-specific RBPs.

NUDT16L1, also called SDOS and TIRR, is a novel RBP that regulates several transcripts encoding for centrosomal proteins and has a key role controlling cilia formation. Cilia are organelles present on eukaryotic cells that plays a role in cell progression. However, for a long time NUDT16L1 has been little studied and novel uncharacterized associations with cancer must be studied^61^.

RBM20 plays a role in the familial cardiomyopathy acting on titin and tropomyosin, two proteins involved in the biomechanics of the striated muscle. It is also associated with fasting glucose regulating insulin damage in cardiac tissues. However, its role in cancer has not yet been demonstrated^62^.

PCDH20, member of subfamily of the cadherin family, is down-regulated in non-small cell lung cancer^63^, nasopharyngeal carcinoma^64^, and hepatocellular carcinoma^65^. The prognostic role of PCDH20 was reported in a recent study: patients with high PCDH20 expression showed a better overall survival than those with low PCDH20 expression in hepatocellular carcinoma^66^. The tumor-suppressor gene PCDH20 through the Wnt/β-catenin signaling pathway acts inhibiting cell proliferation and cell migration^67^.

PCBP3 was associated with favorable prognosis in pancreatic cancer. However, no previous study investigated the molecular mechanism of PCBP3 in carcinogenesis^68^.

Among 29 luminal B-specific RBPs we focused on GSTP1 and RRS1 because previous studies reported an interesting association with BC.

GSTP1 is involved in the drug resistance of tumor cells, including BC. A recent study showed that high expression of GSTP1 can activate the NF-κB signaling pathway in tumor associated macrophages (TAMs) and regulates the expression of IL-6^69^.

RRS1 is a crucial nuclear protein implicated in ribosome biogenesis. It was overexpressed in several human cancers including BC. In addition, elevated RRS1 expression levels were correlated with lymph node metastasis and unfavorable clinical outcome. Some evidence also provided new molecular mechanisms of RRS1 in the proliferation of BC through RPL11/MDM2/p53 pathway^70^.

Among 23 HER2-specific RBPs we identified ALDH18A1 and LASP1 as potential BC biomarkers as they were associated with BC in previous studies. ALDH18A1 is an enzyme implicated in the conversion of glutamine to proline through glutamate. Its over-expression increases proline levels and decreases cell survival in BC as well as reduces reactive oxygen species. ALDH18A1 is also associated with an oncogene, MYC able to regulate cell metabolism and key genes implicated in cancer^71,72^.

LASP1 is a well-known protein that interacts with many proteins regulating tumor cell migration and invasion. A previous study showed that LASP1 binds to Ago2 that plays a key role in BC cell motility in response to CXCR4 activity^73^.

Among 62 basal-specific RBPs we found as interesting biomarkers: SERPINH1 and DKC1.

SERPINH1 plays a key role for the correct folding and secretion of different types of collagen and has previously been associated to cancer progression. Its overexpression is correlated with angiogenesis, migration, and invasion^74^. A previous study demonstrated that SERPINH1 is regulated by miR-148a-5p, a miRNA predictive of unfavorable prognosis^75^.

DKC1 is associated with a poor prognosis in BC. Indeed, patients with a higher expression of DKC1 metastasize more frequently to lymph node than patients with lower DKC1 expression levels. The role of DKC1 in cancer prognosis could be explained by the role of DKC1 in regulating mRNA translation^76^.

Furthermore, in the present study we selected direct interactions involving subtype-specific differentially expressed RBPs and DEGs. Although previous studies demonstrated that numerous lncRNAs are deregulated in different cancer types and RBPs could play a role to the deregulation of lncRNAs^31,77^. in our study we did not find interactions involving differentially expressed lncRNAs and RBPs specific for each subtype. Indeed, the final signature for each subtype is composed of only subtype-specific interacting genes and RBPs. This result can derive by some limitations of our study, such as: (i) lncRNA profiles based on TCGA data, which contain cancer cells and stromal cells could influence the results obtained by differential expression analysis, (ii) the low characterization of lncRNAs in the TCGA data. The characterization of lncRNAs could be more accurate with the new knowledge of data in the future.

However, in our study we found interesting networks consisted of subtype-specific interacting genes and RBPs: a network of only 2 RBPs (RBM20 and PCDH20) and 2 genes (HOXB3 and RASSF7) for luminal A, a network of 21 RBPs and 53 DEGs for luminal B, a HER2-specific network of 14 RBPs and 30 DEGs, and a network of 54 RBPs and 302 DEGs for basal BC. From these networks we investigated the number of RNA targets for each RBP and the number of RBP for each RNA. We found that the number of RBPs per RNA and the number of RNAs per RBP increases with the aggressiveness of the BC molecular subtype. This finding could indicate the key role of the interactions between differentially expressed RBPs and DEGs in the progression of BC. Indeed, luminal A, the less aggressive BC subtype showed a lower number of RNA targets for each RBP and of RBP targets for each RNA. To our knowledge this is the first study that obtained a similar association. Encouraged by the results obtained that demonstrated the specific RBP-RNA interactions for each subtype we validated subtype-specific networks using a machine learning approach on an independent BC dataset from GEO. Overall, we obtained good performances of classification with an accuracy > 0.80 (Table 5). We achieved the best performances from the classification HER2 vs other subtypes (accuracy = 0.96) and basal vs other subtypes (accuracy = 0.96). Overall, given the good results of the classifier we propose the study of these BC subtype-specific interacting biomarkers as potential candidates for differential diagnosis of BC.

Among biomarkers, we found novel RBPs and genes that the survival analysis showed to have a prognostic role.

The low expression of RASSF7, a specific gene of luminal A, plays a prognostic role in BC as it is associated with a poor prognosis. To date, there is not a clear association of RASSF7 with BC.

The survival analysis in this study found that high expression of a specific gene of luminal B, DCTPP1, in a group of 609 BC patients is associated with a poor prognosis respect to 466 BC patients with a low expression. Although previous studies showed its role in DNA damage and genetic instability further studies are needed to investigate its potential therapeutic in BC^78^.

We found that DHRS11, KLC3, NAGS, and TMEM98, specific genes for HER2, are associated with a poor prognosis in BC patients. DHRS11 is implicated in the pathway of cytochrome P450, KLC3 in RHO GTPases activate KTN1M, NAGS in the urea cycle, and TMEM98 in oligodendrocyte differentiation^79^. However, to date there is not a clear association of these genes with BC.

We obtained ABHD14A and ADSSL1 as potential candidate prognostic biomarkers for basal BC. ABHD14A is associated with metabolic disorders of biological oxidation enzymes, and ADSSL1 with Purine ribonucleoside monophosphate biosynthesis^80^.

Although we propose several potential prognostic biomarkers for BC subtypes our study presents some limits. We selected in silico the biomarkers and validated them with a machine learning approach using an independent GEO dataset, and survival analysis. Molecular validation of these biomarkers will be performed in the near future as further studies are needed for translating them to clinical practice.

## Conclusions

In this study, we firstly examined BC subtype-specific DEGs, differentially expressed LNCs and RBPs using BC-TCGA dataset. Then, we investigated the regulatory interactions between RBPs and their target genes in BC subtypes. We found different networks specific for each BC subtype: a network of 2 RBPs (RBM20 and PCDH20) and 2 genes (HOXB3 and RASSF7) for luminal A, a network of 21 RBPs and 53 DEGs for luminal B, a HER2-specific network of 14 RBPs and 30 DEGs, and a network of 54 RBPs and 302 DEGs for basal BC. Overall, the analysis sheds light on the role of RBPs in regulating different BC subtypes and we provided a data exploration analysis to aid future experimental studies. In addition, the analyses in this study suggested some novel prognostic BC biomarkers: RASSF7 for luminal A, DCTPP1 for luminal B, DHRS11, KLC3, NAGS, and TMEM98 for HER2, and ABHD14A and ADSSL1 for basal.

## Supplementary Information

Supplementary Information 1.

Supplementary Information 2.

Supplementary Information 3.

Supplementary Information 4.

Supplementary Information 5.

## Data Availability

The datasets analysed during the current study are available from TCGA portal and GSE58212. This data can be found here: https://portal.gdc.cancer.gov/; https://www.ncbi.nlm.nih.gov/geo/query/acc.cgi?acc=GSE58212.

## Abbreviations

BC: Breast cancer
lncRNAs: Long non-coding RNAs
RBPs: RNA binding-proteins
TCGA: The Cancer genome atlas
NS: Normal samples
DEGs: Differentially expressed genes

## References

[1] Szymiczek A, Lone A, Akbari MR (2020). Molecular intrinsic versus clinical subtyping in breast cancer: a comprehensive review. Clin. Genet..

[2] Bravatà V, Cava C, Minafra L, Cammarata FP, Russo G, Gilardi MC, Castiglioni I, Forte GI (2018). Radiation-induced gene expression changes in high and low grade breast cancer cell types. Int. J. Mol. Sci..

[3] Dai X, Li T, Bai Z, Yang Y, Liu X, Zhan J, Shi B (2015). Breast cancer intrinsic subtype classification, clinical use and future trends. Am. J. Cancer Res..

[4] Fragomeni SM, Sciallis A, Jeruss JS (2018). Molecular subtypes and local-regional control of breast cancer. Surg. Oncol. Clin. N. Am..

[5] Gerdes J, Schwab U, Lemke H, Stein H (1983). Production of a mouse monoclonal antibody reactive with a human nuclear antigen associated with cell proliferation. Int. J. Cancer..

[6] Network CGA (2012). Comprehensive molecular portraits of human breast tumours. Nature.

[7] Perou CM, Sørlie T, Eisen MB, van de Rijn M, Jeffrey SS, Rees CA, Pollack JR, Ross DT, Johnsen H, Akslen LA, Fluge O (2000). Molecular portraits of human breast tumours. Nature.

[8] Sørlie T, Perou CM, Tibshirani R, Aas T, Geisler S, Johnsen H, Hastie T, Eisen MB, van de Rijn M, Jeffrey SS (2001). Gene expression patterns of breast carcinomas distinguish tumor subclasses with clinical implications. Proc. Natl. Acad. Sci. USA.

[9] Pusztai L, Mazouni C, Anderson K, Wu Y, Symmans WF (2006). Molecular classification of breast cancer: limitations and potential. Oncologist..

[10] Goldhirsch, A., Wood, W.C., Coates, A.S., Gelber, R.D., Thürlimann, B., Senn, H.J.; & Panel members. Strategies for subtypes-dealing with the diversity of breast cancer: highlights of the St. Gallen international expert consensus on the primary therapy of early breast cancer 2011. *Ann. Oncol.* **22**(8), 1736–47. doi: 10.1093/annonc/mdr304 (2011) 10.1093/annonc/mdr304 PMC3144634 21709140

[11] Fan, C., Oh, D.S., Wessels, L., Weigelt, B., Nuyten, D.S., Nobel, A.B., van't Veer, L.J., & Perou, C.M. Concordance among gene-expression-based predictors for breast cancer. *N. Engl. J. Med.* **355**(6), 560–9. doi: 10.1056/NEJMoa052933 (2006) 10.1056/NEJMoa052933 16899776

[12] Prat A, Ellis MJ, Perou CM (2011). Practical implications of gene-expression-based assays for breast oncologists. Nat. Rev. Clin. Oncol..

[13] Tsoutsou PG, Vozenin MC, Durham AD, Bourhis J (2017). How could breast cancer molecular features contribute to locoregional treatment decision making?. Crit. Rev. Oncol. Hematol..

[14] Reis-Filho JS, Weigelt B, Fumagalli D, Sotiriou C (2010). Molecular profiling: moving away from tumor philately. Sci. Transl. Med..

[15] Cava, C. *et al.* In silico identification of drug target pathways in breast cancer subtypes using pathway cross-talk inhibition. *J Transl Med*. 10.1186/s12967-018-1535-2 (2018). 10.1186/s12967-018-1535-2 PMC5989433 29871693

[16] Liao GS, Chou YC, Hsu HM, Dai MS, Yu JC (2015). The prognostic value of lymph node status among breast cancer subtypes. Am. J. Surg..

[17] Ignatov A, Eggemann H, Burger E, Ignatov T (2018). Patterns of breast cancer relapse in accordance to biological subtype. J. Cancer Res. Clin. Oncol..

[18] Sims AH, Howell A, Howell SJ, Clarke RB (2007). Origins of breast cancer subtypes and therapeutic implications. Nat. Clin. Pract. Oncol..

[19] Marchese D, de Groot NS, Lorenzo Gotor N, Livi CM, Tartaglia GG (2016). Advances in the characterization of RNA-binding proteins. Wiley Interdiscip. Rev. RNA..

[20] Schmitt AM, Chang HY (2016). Long noncoding RNAs in cancer pathways. Cancer Cell.

[21] Cava C, Bertoli G, Castiglioni I (2019). Portrait of tissue-specific coexpression networks of noncoding RNAs (miRNA and lncRNA) and mRNAs in normal tissues. Comput. Math. Methods Med..

[22] Wang KC, Chang HY (2011). Molecular mechanisms of long noncoding RNAs. Mol. Cell..

[23] Aftabi Y, Ansarin K, Shanehbandi D, Khalili M, Seyedrezazadeh E, Rahbarnia L, Asadi M, Amiri-Sadeghan A, Zafari V, Eyvazi S (2020). Long non-coding RNAs as potential biomarkers in the prognosis and diagnosis of lung cancer: a review and target analysis. IUBMB Life.

[24] Mathias C, Zambalde EP, Rask P, Gradia DF, de Oliveira JC (2019). Long non-coding RNAs differential expression in breast cancer subtypes: what do we know?. Clin. Genet..

[25] Chakravarty D, Sboner A, Nair SS, Giannopoulou E, Li R, Hennig S, Mosquera JM, Pauwels J, Park K, Kossai M (2014). The oestrogen receptor alpha-regulated lncRNA NEAT1 is a critical modulator of prostate cancer. Nat. Commun..

[26] Yang L, Lin C, Jin C, Yang JC, Tanasa B, Li W, Merkurjev D, Ohgi KA, Meng D, Zhang J (2013). lncRNA-dependent mechanisms of androgen-receptor-regulated gene activation programs. Nature.

[27] Hentze MW, Castello A, Schwarzl T, Preiss T (2018). A brave new world of RNA-binding proteins. Nat. Rev. Mol. Cell Biol..

[28] Pereira B, Billaud M, Almeida R (2017). RNA-binding proteins in cancer: old players and new actors. Trends Cancer..

[29] Lujan DA, Ochoa JL, Hartley RS (2018). Cold-inducible RNA binding protein in cancer and inflammation. Wiley Interdiscip. Rev. RNA..

[30] Qin H, Ni H, Liu Y, Yuan Y, Xi T, Li X, Zheng L (2020). RNA-binding proteins in tumor progression. J. Hematol. Oncol..

[31] Jonas K, Calin GA, Pichler M (2020). RNA-binding proteins as important regulators of long non-coding RNAs in cancer. Int. J. Mol. Sci..

[32] Cava C, Colaprico A, Bertoli G, Bontempi G, Mauri G, Castiglioni I (2016). How interacting pathways are regulated by miRNAs in breast cancer subtypes. BMC Bioinform..

[33] Colaprico A, Silva TC, Olsen C, Garofano L, Cava C, Garolini D, Sabedot TS, Malta TM, Pagnotta SM, Castiglioni I, Ceccarelli M, Bontempi G, Noushmehr H (2016). TCGAbiolinks: an R/Bioconductor package for integrative analysis of TCGA data. Nucl. Acids Res..

[34] Benjamini Y, Hochberg Y (1995). Controlling the false discovery rate: a practical and powerful approach to multiple testing. J. R. Stat. Soc. Ser. B Methodol..

[35] Kinsella RJ, Kähäri A, Haider S, Zamora J, Proctor G, Spudich G, Almeida-King J, Staines D, Derwent P, Kerhornou A (2011). Ensembl BioMarts: a hub for data retrieval across taxonomic space. Database (Oxford)..

[36] Volders PJ, Anckaert J, Verheggen K, Nuytens J, Martens L, Mestdagh P, Vandesompele J (2019). LNCipedia 5: towards a reference set of human long non-coding RNAs. Nucl. Acids Res..

[37] Chen H, Boutros PC (2011). VennDiagram: a package for the generation of highly-customizable Venn and Euler diagrams in R. BMC Bioinform..

[38] Vancura A, Lanzós A, Bosch-Guiteras N, Esteban MT, Gutierrez AH, Haefliger S, Johnson R (2021). Cancer LncRNA Census 2 (CLC2): an enhanced resource reveals clinical features of cancer lncRNAs. NAR Cancer..

[39] Lang B, Armaos A, Tartaglia GG (2019). RNAct: protein-RNA interaction predictions for model organisms with supporting experimental data. Nucl. Acids Res..

[40] Marchese, D., Botta-Orfila, T., Cirillo, D., Rodriguez, J.A., Livi, C.M., Fernández-Santiago, R., Ezquerra, M., Martí, M.J., Bechara, E., Tartaglia, G.G.; & Catalan MSA Registry (CMSAR). Discovering the 3' UTR-mediated regulation of alpha-synuclein. *Nucl. Acids Res.* **45**(22), 12888–12903. doi: 10.1093/nar/gkx1048 (2017) 10.1093/nar/gkx1048 PMC5728410 29149290

[41] Cirillo D, Blanco M, Armaos A, Buness A, Avner P, Guttman M, Cerase A, Tartaglia GG (2016). Quantitative predictions of protein interactions with long noncoding RNAs. Nat. Methods..

[42] Bellucci M, Agostini F, Masin M, Tartaglia GG (2011). Predicting protein associations with long noncoding RNAs. Nat. Methods..

[43] Cirillo D, Agostini F, Klus P, Marchese D, Rodriguez S, Bolognesi B, Tartaglia GG (2013). Neurodegenerative diseases: quantitative predictions of protein-RNA interactions. RNA.

[44] Agostini F, Cirillo D, Bolognesi B, Tartaglia GG (2013). X-inactivation: quantitative predictions of protein interactions in the Xist network. Nucl. Acids Res..

[45] Uhlen M, Zhang C, Lee S, Sjöstedt E, Fagerberg L, Bidkhori G, Benfeitas R, Arif M, Liu Z, Edfors F, Sanli K, von Feilitzen K, Oksvold P, Lundberg E, Hober S, Nilsson P, Mattsson J, Schwenk JM, Brunnström H, Glimelius B, Sjöblom T, Edqvist PH, Djureinovic D, Micke P, Lindskog C, Mardinoglu A, Ponten F (2017). A pathology atlas of the human cancer transcriptome. Science.

[46] Jager KJ, van Dijk PC, Zoccali C, Dekker FW (2008). The analysis of survival data: the Kaplan-Meier method. Kidney Int..

[47] Max Kuhn caret: Classification and Regression Training. Accessed on 2 Feb 2020

[48] Gendrel AV, Heard E (2011). Fifty years of X-inactivation research. Development.

[49] Chiu, H.S., Somvanshi, S., Patel, E., Chen, T.W., Singh, V.P., Zorman, B., Patil, S.L., Pan, Y., Chatterjee, S.S.; Cancer Genome Atlas Research Network, Sood, A.K., Gunaratne, P.H., & Sumazin, P. Pan-cancer analysis of lncRNA regulation supports their targeting of cancer genes in each tumor context. *Cell Rep.* **23**(1), 297–312.e12. doi: 10.1016/j.celrep.2018.03.064 (2018) 10.1016/j.celrep.2018.03.064 PMC5906131 29617668

[50] Katopodis P, Dong Q, Halai H, Fratila CI, Polychronis A, Anikin V, Sisu C, Karteris E (2020). In silico and in vitro analysis of lncRNA XIST reveals a panel of possible lung cancer regulators and a five-gene diagnostic signature. Cancers (Basel)..

[51] Wang H, Huang Y, Yang Y (2020). LncRNA PVT1 regulates TRPS1 expression in breast cancer by sponging miR-543. Cancer Manag. Res..

[52] You Z, Xu S, Pang D (2020). Long noncoding RNA PVT1 acts as an oncogenic driver in human pan-cancer. J. Cell Physiol..

[53] Alvarez ML, Khosroheidari M, Eddy E, Kiefer J, DiStefano JK (2016). Correction: role of MicroRNA 1207–5P and its host gene, the long non-coding RNA Pvt1, as mediators of extracellular matrix accumulation in the kidney: implications for diabetic nephropathy. PLoS ONE.

[54] Fan D, Qiu B, Yang XJ, Tang HL, Peng SJ, Yang P, Dong YM, Yang L, Bao GQ, Zhao HD (2020). LncRNA SNHG8 promotes cell migration and invasion in breast cancer cell through miR-634/ZBTB20 axis. Eur. Rev. Med. Pharmacol. Sci..

[55] Tian X, Liu Y, Wang Z, Wu S (2020). lncRNA SNHG8 promotes aggressive behaviors of nasopharyngeal carcinoma via regulating miR-656-3p/SATB1 axis. Biomed. Pharmacother..

[56] Dobre EG, Dinescu S, Costache M (2020). Connecting the missing dots: ncRNAs as critical regulators of therapeutic susceptibility in breast cancer. Cancers (Basel)..

[57] Xu S, Wang Q, Kang Y, Liu J, Yin Y, Liu L, Wu H, Li S, Sui S, Shen M (2020). Long noncoding RNAs control the modulation of immune checkpoint molecules in cancer. Cancer Immunol. Res..

[58] Wang L, Luan T, Zhou S, Lin J, Yang Y, Liu W, Tong X, Jiang W (2019). LncRNA HCP5 promotes triple negative breast cancer progression as a ceRNA to regulate BIRC3 by sponging miR-219a-5p. Cancer Med..

[59] Jiang H, Li X, Wang W, Dong H (2020). Long non-coding RNA SNHG3 promotes breast cancer cell proliferation and metastasis by binding to microRNA-154-3p and activating the notch signaling pathway. BMC Cancer.

[60] Elton TS, Selemon H, Elton SM, Parinandi NL (2013). Regulation of the MIR155 host gene in physiological and pathological processes. Gene.

[61] Taniguchi-Ponciano K, Huerta-Padilla V, Baeza-Xochihua V, Ponce-Navarrete G, Salcedo E, Gomez-Apo E, Chavez-Macias L, Aviles-Duran J, Ruiz-Sanchez H, Valdivia A (2019). Revisiting the genomic and transcriptomic landscapes from female malignancies could provide molecular markers and targets for precision medicine. Arch. Med. Res..

[62] Avolio R, Järvelin AI, Mohammed S, Agliarulo I, Condelli V, Zoppoli P, Calice G, Sarnataro D, Bechara E, Tartaglia GG (2018). Protein syndesmos is a novel RNA-binding protein that regulates primary cilia formation. Nucl. Acids Res..

[63] Liu J, Carnero-Montoro E, van Dongen J, Lent S, Nedeljkovic I, Ligthart S, Tsai PC, Martin TC, Mandaviya PR, Jansen R (2019). An integrative cross-omics analysis of DNA methylation sites of glucose and insulin homeostasis. Nat. Commun..

[64] Imoto I, Izumi H, Yokoi S, Hosoda H, Shibata T, Hosoda F, Ohki M, Hirohashi S, Inazawa J (2006). Frequent silencing of the candidate tumor suppressor PCDH20 by epigenetic mechanism in non-small-cell lung cancers. Cancer Res..

[65] Chen T, Long B, Ren G, Xiang T, Li L, Wang Z, He Y, Zeng Q, Hong S, Hu G (2015). Protocadherin20 acts as a tumor suppressor gene: epigenetic inactivation in nasopharyngeal carcinoma. J. Cell Biochem..

[66] Lv J, Zhu P, Yang Z, Li M, Zhang X, Cheng J, Chen X, Lu F (2015). PCDH20 functions as a tumour-suppressor gene through antagonizing the Wnt/beta-catenin signalling pathway in hepatocellular carcinoma. J. Viral Hepat..

[67] Wu Y, Zheng S, Yao J, Li M, Yang G, Zhang N, Zhang S, Zhong B (2017). Decreased expression of protocadherin 20 is associated with poor prognosis in hepatocellular carcinoma. Oncotarget.

[68] Ger M, Kaupinis A, Petrulionis M, Kurlinkus B, Cicenas J, Sileikis A, Valius M, Strupas K (2018). Proteomic identification of FLT3 and PCBP3 as potential prognostic biomarkers for pancreatic cancer. Anticancer Res..

[69] Dong X, Sun R, Wang J, Yu S, Cui J, Guo Z, Pan X, Sun J, Yang J, Pan LL (2020). Glutathione S-transferases P1-mediated interleukin-6 in tumor-associated macrophages augments drug-resistance in MCF-7 breast cancer. Biochem. Pharmacol..

[70] Song J, Ma Z, Hua Y, Xu J, Li N, Ju C, Hou L (2018). Functional role of RRS1 in breast cancer cell proliferation. J. Cell Mol. Med..

[71] Craze ML, Cheung H, Jewa N, Coimbra NDM, Soria D, El-Ansari R, Aleskandarany MA, Wai Cheng K, Diez-Rodriguez M, Nolan CC (2018). MYC regulation of glutamine-proline regulatory axis is key in luminal B breast cancer. Br. J. Cancer..

[72] Grinde MT, Hilmarsdottir B, Tunset HM, Henriksen IM, Kim J, Haugen MH, Rye MB, Mælandsmo GM, Moestue SA (2019). Glutamine to proline conversion is associated with response to glutaminase inhibition in breast cancer. Breast Cancer Res..

[73] Tilley AMC, Howard CM, Sridharan S, Subramaniyan B, Bearss NR, Alkhalili S, Raman D (2020). The CXCR4-dependent LASP1-Ago2 interaction in triple-negative breast cancer. Cancers (Basel)..

[74] Strack E, Rolfe PA, Fink AF, Bankov K, Schmid T, Solbach C, Savai R, Sha W, Pradel L, Hartmann S, Brüne B, Weigert A (2020). Identification of tumor-associated macrophage subsets that are associated with breast cancer prognosis. Clin. Transl. Med..

[75] Kawagoe K, Wada M, Idichi T, Okada R, Yamada Y, Moriya S, Okubo K, Matsushita D, Arigami T, Kurahara H (2020). Regulation of aberrantly expressed SERPINH1 by antitumor miR-148a-5p inhibits cancer cell aggressiveness in gastric cancer. J. Hum. Genet..

[76] Guerrieri AN, Zacchini F, Onofrillo C, Di Viggiano S, Penzo M, Ansuini A, Gandin I, Nobe Y, Taoka M, Isobe T (2020). DKC1 overexpression induces a more aggressive cellular behavior and increases intrinsic ribosomal activity in immortalized mammary gland cells. Cancers (Basel)..

[77] Zhang Q, Wei Y, Yan Z, Wu C, Chang Z, Zhu Y, Li K, Xu Y (2017). The characteristic landscape of lncRNAs classified by RBP-lncRNA interactions across 10 cancers. Mol. Biosyst..

[78] Niu M, Shan M, Liu Y, Song Y, Han JG, Sun S, Liang XS, Zhang GQ (2021). DCTPP1, an oncogene regulated by miR-378a-3p, promotes proliferation of breast cancer via DNA repair signaling pathway. Front. Oncol..

[79] Stelzer G, Rosen N, Plaschkes I, Zimmerman S, Twik M, Fishilevich S, Stein TI, Nudel R, Lieder I, Mazor Y, Kaplan S, Dahary D, Warshawsky D, Guan-Golan Y, Kohn A, Rappaport N, Safran M, Lancet D (2016). The GeneCards suite: from gene data mining to disease genome sequence analyses. Curr. Protoc. Bioinform..

[80] Jassal B, Matthews L, Viteri G, Gong C, Lorente P, Fabregat A, Sidiropoulos K, Cook J, Gillespie M, Haw R, Loney F, May B, Milacic M, Rothfels K, Sevilla C, Shamovsky V, Shorser S, Varusai T, Weiser J, Wu G, Stein L, Hermjakob H, D'Eustachio P (2020). The reactome pathway knowledgebase. Nucl. Acids Res..

