# Supplementary material for: Identification of long non-coding RNAs and RNA binding proteins in breast cancer subtypes
Source: Sci Rep. 2022 Jan 13;12:693. doi: 10.1038/s41598-021-04664-z (PMC8758778; doi:10.1038/s41598-021-04664-z)
Supplement: Supplementary file 1 — Supplementary Information 1. [file 41598_2021_4664_MOESM1_ESM.docx]

**Supplementary Information**

Supplementary file 1: shows the list of DEGs for each subtype.

Supplementary file 2: shows the list of RBPs for each subtype.

Supplementary file 3: Distribution of Z-score used to assess overall binding ability between 5980 RNAs and 281 RBPs.

Supplementary file 4: shows the distribution of differentially expressed interacting biomarkers in at least one BC subtype.
