# Supplementary material for: Identification of long non-coding RNAs and RNA binding proteins in breast cancer subtypes
Source: Sci Rep. 2022 Jan 13;12:693. doi: 10.1038/s41598-021-04664-z (PMC8758778; doi:10.1038/s41598-021-04664-z)
Supplement: Supplementary file 4 — Supplementary Information 4. [file 41598_2021_4664_MOESM4_ESM.pptx]

## Slide 1
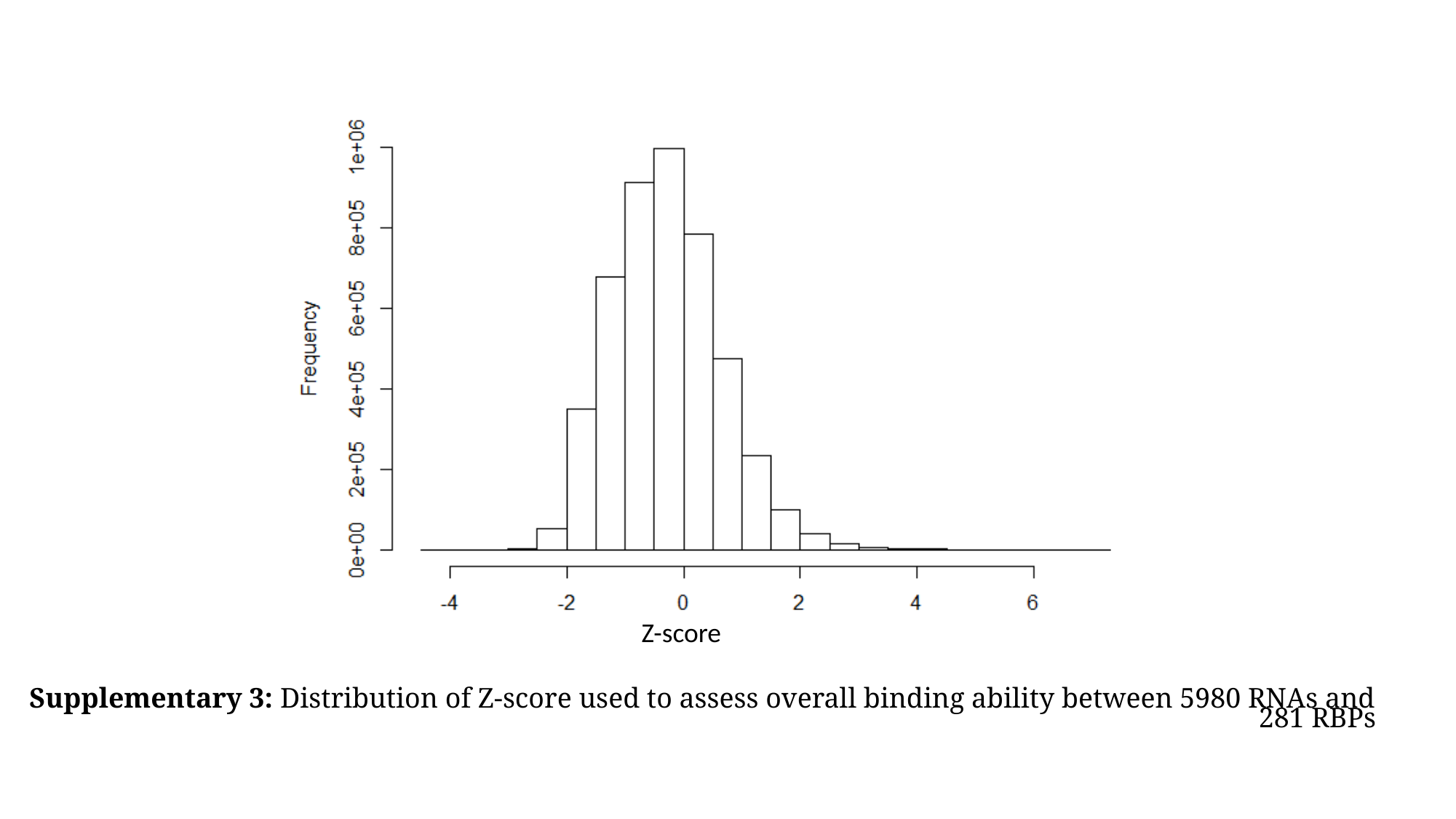

Z-score
Supplementary 3: Distribution of Z-score used to assess overall binding ability between 5980 RNAs and 281 RBPs
